# Disease tolerance as immune defense strategy in bats: One size fits all?

**DOI:** 10.1371/journal.ppat.1012471

**Published:** 2024-09-05

**Authors:** Gang Pei, Anne Balkema-Buschmann, Anca Dorhoi

**Affiliations:** 1 Institute of Immunology, Friedrich-Loeffler-Institut, Federal Research Institute for Animal Health, Greifswald-Insel Riems, Germany; 2 Institute of Novel and Emerging Infectious Diseases, Friedrich-Loeffler-Institut, Federal Research Institute of Animal Health, Greifswald-Insel Riems, Germany; 3 Faculty of Mathematics and Natural Sciences, University of Greifswald, Greifswald, Germany; University of Queensland, AUSTRALIA

## Abstract

Bats are natural reservoirs for zoonotic pathogens, yet the determinants of microbial persistence as well as the specific functionality of their immune system remain largely enigmatic. Their propensity to harbor viruses lethal to humans and/or livestock, mostly in absence of clinical disease, makes bats stand out among mammals. Defending against pathogens relies on avoidance, resistance, and/or tolerance strategies. In bats, disease tolerance has recently gained increasing attention as a prevailing host defense paradigm. We here summarize the current knowledge on immune responses in bats in the context of infection with zoonotic agents and discuss concepts related to disease tolerance. Acknowledging the wide diversity of bats, the broad spectrum of bat-associated microbial species, and immune-related knowledge gaps, we identify research priorities necessary to provide evidence-based proofs for disease tolerance in bats. Since disease tolerance relies on networks of biological processes, we emphasize that investigations beyond the immune system, using novel technologies and computational biology, could jointly advance our knowledge about mechanisms conferring bats reservoir abilities. Although disease tolerance may not be the “one fit all” defense strategy, deciphering disease tolerance in bats could translate into novel therapies and inform prevention of spillover infections to humans and livestock.

## 1. Introduction

Many emerging zoonotic infections originate from wildlife, and the vast majority are caused by viral pathogens. The richness of zoonotic pathogens associated with bats exceeds the richness detected in many other mammalian orders [1–3], and the association between bats and the high-impact viruses is exceptional [4]. Bats have been shown to harbor Marburg virus (MARV), Nipah virus (NiV), and Hendra virus (HeV), agents that trigger lethal infections in humans and livestock [5]. They also maintain multiple coronaviruses posing substantial threats to humans and farm animals, including severe acute respiratory syndrome coronavirus (SARS-CoV), SARS-CoV-2-related viruses, and swine acute diarrhea syndrome coronavirus (SADS-CoV) [5–7]. Moreover, bats have been postulated to act as reservoir hosts for numerous other viruses, as deduced from molecular detection of virus sequences in bat samples, including species from the genus *Orthoebolavirus* (with Ebola virus (EBOV) as the most relevant representative of the species *Orthoebolavirus zairense*) and the subgenus *Merbecovirus* (with Middle East respiratory syndrome coronavirus (MERS-CoV) as the most prominent representative of the species *Betacoronavirus cameli*) [8,9]. Numerous studies have demonstrated that aberrant immune activation contributes to the pathogenesis of diseases caused by the abovementioned viruses in humans [10,11], while infected bats display no or minimal clinical signs [12–17]. How can bats cope with viruses lethal for other mammals? How can bats harbor multiple zoonotic pathogens? Several hypotheses have been advanced to explain the lack of clinical manifestations in bats. Asymptomatic infection has been linked to “flight as fever” [18], constitutive antiviral immunity or “always on” mechanisms [19], and dampened inflammation [20,21]. More recently, disease tolerance has been proposed as an immune defense strategy operative in bats [22–24].

Humans and animals defend against infections using 3 main strategies: avoidance, resistance, and tolerance [25]. These terms will be used in the following and definitions are provided to avoid confusions and inaccurate terminology interchange. Avoidance restricts the infection by limiting the host exposure to pathogens. As an example, *Caenorhabditis elegans* avoids the pathogenic bacteria *Serratia marcescens* by detecting the bacterial product serrawettin W2 via chemosensory neurons [26]. Disease resistance relies on immune responses that kill or eliminate the pathogens, i.e., antimicrobial, antiviral, antifungal, and antiparasitic effectors and pathways [25]. The vast majority of research attention has been given to this modality of immune defense. Disease tolerance maintains host fitness by minimizing or offsetting health costs or tissue damage caused by infection, while it does not directly impact on the pathogen burden [25,27,28]. A disease-tolerant host can maintain a high pathogen burden without suffering severe illness or death. For example, different mouse strains display variable tolerance to *Plasmodium* infection, and disease severity is dissociated from pathogen burden [27]. Notably, resistance and tolerance to disease are not mutually exclusive and are often interrelated. Resistance may operate in coordination with tolerance to protect the host against infection or revert organismal functions to the physiological state [28]. Of note, disease tolerance differs from immunological tolerance or immune tolerance, which means unresponsiveness to self-antigens, and it also differs from endotoxin tolerance, which signifies repressed responses to lipopolysaccharides induced by repeated exposure. To avoid any confusion, it must also be indicated that resilience is not similar to disease tolerance. Resilience denotes the capacity of an individual to recover from illness and can be modulated by both disease resistance and tolerance.

Disease tolerance has been considered as an integral host response of plants to microbes for a long time [29]. Disease tolerance relies on the mechanisms alleviating the tissue damage induced by both pathogens themselves and/or inflammation, including neutralization and detoxification, cellular stress responses, metabolic adaptation, inflammatory control, and tissue repair [25,28]. Its relevance in mammals was only demonstrated less than 2 decades back [27]. Disease tolerance has been postulated for bats [22,24] and currently represents a field of intense scientific research focusing primarily on the control of inflammation. Considering the relevance of inflammation for tissue damage and host fitness, we discuss unique features of inflammation in bats, which may confer them reservoir abilities. In addition to inflammation control, we elaborate on disease tolerance mechanisms known from experimental studies and discuss evidence for and translatability to bats. We assemble a catalogue of questions about disease tolerance in bats and conclude on its potential role as the major host defense strategy in bats. The translational benefits of bat-specific mechanisms of disease tolerance to human patients have been recently discussed [23] and are beyond the scope of this review.

## 2. Bat-borne zoonotic infections

Over 1,400 species of bats have been identified worldwide, representing more than 20% of classified mammal diversity [30]. The vast diversity of viruses identified in bat population has been reviewed elsewhere [31,32]. Given substantial immunological variations among bat species, we particularly focus on immune responses to zoonotic pathogens in their corresponding bat reservoirs. We summarized the current knowledge on infections with these agents in experimentally infected bats (Table 1) and discuss specific host response patterns in the context of bat and virus diversity. To avoid any misconception, we clarify the terms “natural host” and “reservoir host,” as these are frequently used in the context of infectious diseases. Natural host denotes host species as the ecological niche that can maintain a viral population or metapopulation and is frequently employed by virologists [33]. Reservoir host is an epidemiology concept in which a population or species can support the replication and circulation of a pathogen in long term, finally facilitating transmission to the susceptible species [34]. Natural hosts could be reservoir hosts, yet not all natural hosts act as reservoir hosts. It is difficult to establish whether a specific population or species is a reservoir for a particular pathogen, which requires evidence from ecology, epidemiology, virology, immunology, and pathology. Hence, in the following, we will use natural hosts instead of reservoir hosts if evidences are insufficient to conclude on the reservoir host role.

**Table 1:** Bat-borne zoonotic viruses, their potential reservoir hosts, and clinical signs in bats upon experimental challenge.

Virus family	Virus	Reservoir host	Clinical signs	References
*Paramyxoviridae*	Hendra virus (HeV)	*Pteropus poliocephalus*, *Pteropus conspicillatus*, *Pteropus scapulatus*, *Pteropus alecto*	Subclinical disease in *P*. *poliocephalus* (s.c. and i.n.), subclinical disease in pregnant *P*. *poliocephalus* (s.c.).	[12,13]
	Nipah virus (NiV)	*Pteropus hypomelanus*, *Pteropus vampyrus*	Subclinical disease in *P*. *poliocephalus* (s.c.)	[14,35]
	Sosuga virus (SOSV)	*Rousettus aegyptiacus*	Mild signs with viral replication, persistence. and shedding	[36]
*Coronaviridae*	SARS-CoV ancestor	*Rhinolophus* spp. (?)	N.D.	[37]
	SARS-CoV-2 ancestor	*Rhinolophus* spp. (?)	N.D.	[38]
*Filoviridae*	Marburg virus (MARV)	*Rousettus aegyptiacus*	No clinical signs with viral replication, persistence, viral shedding, and transmission	[15–17,39]
*Orthonairoviridae*	Kasokero virus (KASV)	*Rousettus aegyptiacus*	No clinical signs, long-lasting viremia	[40]
*Orthomyxoviridae*	Influenza A virus H9N2	*Rousettus aegyptiacus*	No clinical signs, rapid clearance, development of neutralizing antibodies	[41]
*Rhabdoviridae*	Rabies virus (RABV)	Various insectivorous, haematophagous, and frugivorous bats, i.e., *Tadarida brasiliensis*,	Clinical rabies disease in *T*. *braziliensis* (i.c., i.m., or s.c.)	[42,43]
	European bat 1 lyssavirus (EBLV-1)	*Eptesicus serotinus*, *E*. *isabellinus*	Rabies-like disease in *E*. *serotinus* (i.c., i.m.)	[44,45]
	European bat 2 lyssavirus (EBLV-2)	*Myotis daubentonii*, *Myotis dasycneme*	Clinical rabies disease in *M*. *daubentonii* (i.c.), no infection (i.m., i.n.)	[46]
	Lagos bat virus (LBV)	*Eidolon helvum*	Fatal clinical rabies infection	[47,48]

i.c., intracerebral; i.m., intramuscular; i.n., intranasal; N.D., not determined; s.c., subcutaneous.

## 3. Immune responses and limited inflammation upon virus infection in bats

Aberrant inflammatory responses contribute to the pathogenesis of diseases caused by NiV, HeV, EBOV, SARS-CoV, and SARS-CoV-2 in humans [10,11]. In contrast, experimental challenge of the corresponding reservoir hosts with HeV [12], NiV [14], or MARV [15–17] led to subclinical disease or asymptomatic infection. The discrepancy in disease outcome may be due to a blunt proinflammatory response in bats compared to disease-susceptible hosts. For example, MARV-infected *Rousettus aegyptiacus* bats up-regulate canonical antiviral pathways, but no other proinflammatory genes [22,49]. Such observations propelled disease tolerance as host defense mechanism, which may explain bats’ unique reservoir potential [24]. Accordingly, bats may support the virus replication as humans do, while restraining proinflammatory responses and subsequent immunopathology. However, bats do develop disease upon infection with certain bat pathogens. For instance, infection with a high dose of Tacaribe virus leads to substantial mortality with pathology involving multiple organs in *Artibeus jamaicensis*, a natural host of Tacaribe virus [50]. *Pseudogymnoascus destructans*, the causative agent of white-nose syndrome, triggers skin lesions and massive mortality of hibernating bats in North America [51]. These examples suggest that inflammation control fails to confer disease tolerance to some microbes. In the following section, we summarize the features of the bat immune system and discuss their relevance for disease tolerance in bats in the context of zoonotic viruses.

### 3.1. The interferon system: Induction and its regulation

#### 3.1.1. Largely conserved RNA sensing

In humans, activation of endosomal Toll-like receptor 3 (TLR3), TLR7, and TLR8 triggers the release of the type I interferon (IFN-I) in response to RNA recognition [52]. TLR3 recognizes double-stranded RNA (dsRNA), while TLR7 and TLR8 detect single-stranded RNA (ssRNA). Upon dsRNA engagement, TIR domain-containing adaptor protein (TRIF) is recruited to TLR3 and induces interferon regulatory factor 3 (IRF3) activation and subsequent IFN-I production [52]. Activated TLR7 or TLR8 recruits myeloid differentiation primary response 88 (Myd88) and triggers IRF7- or IRF3/7-mediated IFN-I induction, respectively [52]. Retinoic acid-inducible gene I (RIG-I) or melanoma differentiation-associated protein 5 (MDA5) are pattern-recognition receptors (PRR) detecting cytosolic dsRNA [52]. Upon ligand binding, they recruit mitochondrial antiviral signaling protein (MAVS) and activate IRF3-dependent IFN responses [52]. Analysis of TLR7 and TLR8 protein sequences from 10 bat species covering Yangochiroptera and Yinpterochiroptera has demonstrated positive selection of TLR7 and TLR8 [53]. All the positive selection sites are located in the leucine-rich repeat (LRR) domains, likely reflecting viral adaptation in bats. In contrast, bat TLR3 is under strong negative selection [53] and cells from *Eptesicus fuscus* engage rather TLR3 over RIG-I or MDA5 for IFN-I production [54]. Molecular characterization of RIG-I and MDA5 in *Pteropus alecto* suggests that they resemble those from other mammals [55]. Overexpression of *Tadarida brasiliensis* MDA5 induces IFN-I-dependent inhibition of vesicular stomatitis virus (VSV) replication, further suggesting conserved function of bat MDA5 [56]. Comparison of human RIG-I with counterparts from *Myotis daubentonii* and *Rousettus aegyptiacus*, representing Yangochiroptera and Yinpterochiroptera, respectively, has demonstrated that all 3 RIG-I orthologs similarly induce IFN-I in response to viral RNA [57]. Taken together, the functions of the bat RNA sensors TLR3, RIG-I, and MDA5 are conserved, and the roles of bat TLR7 and TLR8 need to be further investigated. RNA sensing seems to be central for IFN-I production and likely disease resistance in bats, yet the involvement of individual RNA sensors in disease tolerance remains questionable.

#### 3.1.2. Dampened DNA sensing

During flight, bats exhibit high body temperature that can reach 42°C [58] and elevated metabolic rates that may generate excessive reactive oxygen species (ROS) [59]. Both heat shock and oxidative stress can result in DNA damage and DNA release into the cytosol [60,61]. To avoid cell and tissue injury, bats have likely developed mechanisms to cope with the DNA damage. Comparative analysis of bat and other mammals’ genomes has demonstrated that genes involved in DNA damage repair are under positive selection in bats, likely contributing to enhanced and timely DNA damage repair [62]. The cytosolic PRR absent in melanoma 2 (AIM2) and interferon gamma-inducible protein 16 (IFI16) mediate inflammasome activation by sensing DNA of microbial or host origin [63–67]. The Pyrin and hematopoietic interferon-inducible nuclear (HIN) domain-containing (PYHIN) gene family, including AIM2, IFI16, Pyhin1, and myeloid nuclear differentiation antigen (MNDA) (a paralog of Pyhin1), is completely absent in genomes of *Pteropus alecto* and *Myotis davidii*, representative bats of Yinpterochiroptera and Yangochiroptera suborders, respectively [62]. This observation has been extended to additional 37 bats species [68,69] and indicates a general mechanism to suppress cytosolic DNA-induced inflammasome immunopathology in bats. This evolutionary adaptation possibly restricts immune activation due to flight-associated DNA damage or infection with DNA viruses and intracellular bacteria.

**Fig 1:**
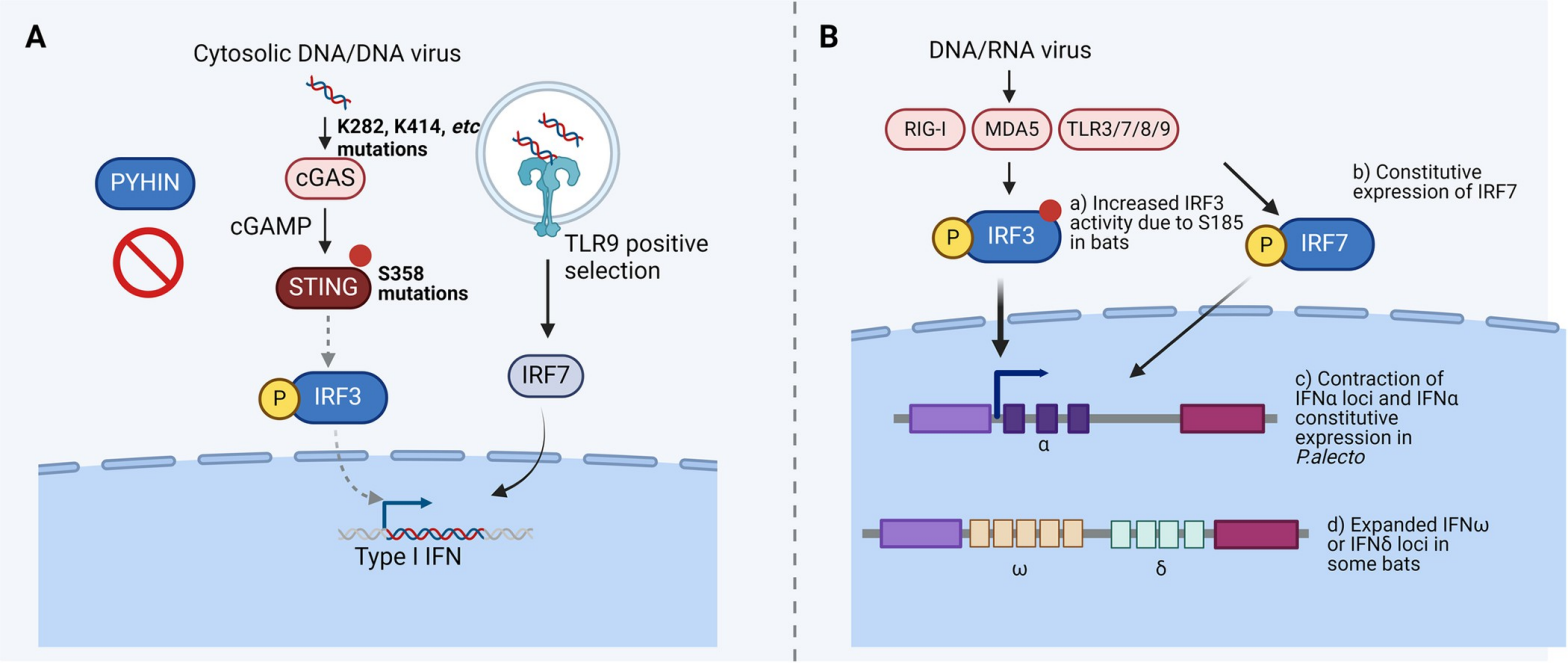
Unique features of IFN signaling in bats. **(A)** Reduction of DNA immune sensing in bats. Mutations (for instance, K282, K414) in the catalytic domain of cGAS and a unique mutation corresponding to S358 of STING results in impaired cytosolic DNA-induced IFN signaling. TLR9 in bats is under high positive selection affecting sites in the LRR domain; however, its relevance in DNA sensing remains elusive. In addition, the PYHIN gene family that senses cytosolic DNA and activates inflammasome activation is completely lost in bats. **(B)** Elevated IFN signaling in bats. (a) Positive selection of the residue at position 185 in bat IRF3 results in increased IRF3 activity. (b) IRF7 is constitutively expressed in *Pteropus alecto*, likely contributing to the high constitutive expression of IFN-α. (c) The loci of IFN-α are contracted with a constitutive expression under basal condition in *Pteropus alecto*. (d) The loci of IFNω and IFN-δ are expanded in some bats. The figure was generated using the illustration software BioRender (BioRender.com). cGAS, cyclic GMP-AMP synthase; HIN, hematopoietic interferon-inducible nuclear; IFN, interferon; IRF, interferon regulatory transcription factor; LRR, leucine-rich repeat; MDA5, melanoma differentiation-associated protein 5; PYHIN, Pyrin and HIN domain; RIG-I, retinoic acid-inducible gene I; STING, stimulator of interferon genes; S358, serine at position 358; TLR, Toll-like receptor.

Other PRR recognizing DNA, such as DNA-dependent activator of IFN-regulatory factor (DAI), DDX41, cyclic GMP-AMP synthase (cGAS), and TLR9, initiate IFN-I responses in humans and mice [70]. Among them, cGAS has emerged as the major sensor for cytosolic DNA [71]. Upon activation, cGAS produces cyclic GMP-AMP (cGAMP), which binds to and activates stimulator of interferon genes (STING). Subsequently, STING recruits TANK-binding kinase 1 (TBK1) and triggers IRF3 phosphorylation to initiate IFN-I production [71]. In the bat family Pteropodidae, cGAS has been identified as one of the positively selected genes. Of the 16 positive selection sites, 14 are located in the catalytic domain [72]. Mutations of 2 such residues (K282 and K414) lead to significantly reduced activity of human cGAS [73,74], indicating impaired cGAS activity in pteropodids (Fig 1A). Consistently, comprehensive alignment of STING from 30 bat species has uncovered a unique mutation corresponding to serine at position 358 (S358) [75] (Fig 1A). S358 of human STING is required for IRF3 activation [76]. Stimulation of splenocytes from the Chinese horseshoe bat (*Rhinolophus sinicus*) with cGAMP induces much lower levels of IFNβ and interferon-stimulated genes (ISG) compared to mice [75]. Thus, dampened DNA-induced IFN-I responses resulting from substitution of the S358 residue in bat STING may represent a general mechanism employed by bats to control inflammation.

TLR9 recognizes DNA containing unmethylated CpG motifs present in bacterial, viral, and mitochondrial DNA [70]. Following DNA recognition, TLR9 induces IRF7-dependent IFN-I release and NF-κB-dependent proinflammatory cytokine production [70]. Phylogenetic analysis has revealed that bat TLR9 forms a monophyletic clade apart from all other eutherian mammals [53]. TLR9 is also under high positive selection affecting sites located in the LRR domain, which mediates ligand interaction. These evolutionary traits likely impact on pathogen recognition and downstream signaling [53,77] (Fig 1A). In the genomes of papillomaviruses identified in *Tadarida brasiliensis*, CpG sites, particularly ACGT, a known TLR9 recognition motif, are significantly depleted, further supporting viral evolution toward TLR9 avoidance [78]. Although TLR9 is mainly expressed in plasmacytoid dendritic cells (pDCs) and B cells [79], it is also detected on the plasma membrane of human and murine red blood cells (RBC). CpG-containing DNA binds to RBC via TLR9, thus triggering erythrophagocytosis and IFN-I responses [80]. RBC from *Eptesicus fuscus* express TLR9 and bind CpG-containing DNA [81]. Interestingly, crenated and poikilocytic RBC resembling human RBC during acute inflammation [80] have been observed in different bat species [81,82]. Whether morphology is linked to DNA immune sensing and to constitutive RBC activation in bats remains to be evaluated. In summary, limited immunopathology resulting from restriction of IFN-I induction and inflammasome activation in response to DNA, in combination with enhanced DNA damage repair may contribute to disease tolerance during viral infections.

#### 3.1.3. Type I interferons (IFN-I)

IFNs are a family of cytokines that trigger an antiviral state of the host by inducing the expression of ISG [83]. According to the amino acid composition and their receptors, IFNs are categorized into 3 distinct groups, designated IFN-I, type II (IFN-II), and type III IFN (IFN-III) [83,84]. IFN-I, such as IFNα, IFNβ, IFNκ, and IFNω, are produced by diverse cell types upon viral infection [83]. IFN-I genomic locus in black flying fox (*Pteropus alecto*) contains 10 IFN-I loci with only 3 IFNα genes [19], and in the genome of *Myotis lucifugus*, IFNα locus is absent [85]. However, the contraction of IFNα locus has not been observed in *Pteropus vampyrus* and *Rousettus aegyptiacus* [85,86]. IFNα is constitutively expressed in diverse tissues and cells from *Pteropus alecto* and from the lesser short-nosed fruit bat (*Cynopterus brachyotis*) (Fig 1B). IFNα has been measured in plasma from *Pteropus rodricensis* and *Rousettus aegyptiacus*, suggesting ubiquitous release of IFNα in these bats at steady state. Bat IFNα3 induces ISGs expression and antiviral activity against Pteropine orthoreovirus Nelson Bay strain (NB), confirming functionality of IFNα in bats [19]. Other studies have found no evidence of basal expression of IFN-I in *Rousettus aegyptiacus* albeit expansion of the IFN-I locus in this bat [86]. Loci encoding for IFNω are expanded in various bat species, including *Rousettus aegyptiacus*, *Pteropus vampyrus*, and *Myotis lucifugus* [85,86]. Upon Sendai virus infection, IFNα, IFNβ, and IFNω expression is induced in immortalized *Rousettus aegyptiacus* cells [86]. Both IFNω4 and IFNω9 display antiviral activities, with IFNω9 triggering more potent restriction of viral replication [87]. IFNω from the European serotine bat, *Eptesicus serotinus*, also demonstrates strong antiviral activity against EBLV-1/EBLV-2 [88]. IFN-δ, only identified in pig, sheep, and horse, is expressed in trophoblast during early pregnancy [89,90]. The IFN-δ locus in *Pteropus vampyrus* and *Myotis lucifugus* is expanded, possibly contributing to prevention of perinatal transmission of viruses [85].

IFN-I induction pathways are conserved in bats. IRF3 is a critical transcription factor mediating IFN-I gene expression upon virus detection [83]. It is also crucial for IFNβ induction in cells from *Eptesicus fuscus* in response to poly(I:C) and MERS-CoV stimulation [91]. Sequence analysis of bat IRF3 across Yinpterochiroptera and Yangochiroptera suborder has identified positive selection of the residue at position 185 in bat IRF3, which is essential for the antiviral activity in *Eptesicus fuscus* and *Pteropus alecto* cells [92]. However, IRF3 from many bat species have different amino acids instead of serine at this position [92]. Whether these mutations have the same effect needs to be clarified. IFN-I strongly activates the expression of IRF7, which, in turn, triggers IFNα expression in a positive feedback loop [93]. Interestingly, the expression of IRF7 is constitutively expressed among all tissues from *Pteropus alecto*, and its expression is further induced by poly(I:C) stimulation or IFN-I. Bat IRF7 interacts with Myd88 and drives IFNα promoter activation in *Pteropus alecto*, which could explain the constitutive expression of IFNα in this bat [19,94]. Basal expression levels of IRF1/3/7 are higher in tissues from *Pteropus alecto* compared to mice. Intriguingly, they promote the expression of different subsets of ISG under basal condition and upon viral infections and are nonredundant for controlling MERS-CoV and influenza A virus (IAV), but not Herpes simplex virus (HSV) replication [95] (Fig 1B).

IFN-I receptor (IFNAR1/2) is a dimeric molecule and IFNAR1 is positively selected in bats, but its relevance in IFN signaling is not fully elucidated [62,86,96]. Once bound to their receptors, IFN-I activate receptor-associated tyrosine kinase 2 (TYK2) and Janus-activated kinase 1 (JAK1) and subsequently induce the phosphorylation of signal transducer and activator of transcription 1 (STAT1) and STAT2 [83]. This leads to the formation of STAT1-STAT2-IRF9 transcriptional complexes (also known as ISGF3), which, in turn, translocate to the nucleus and initiate the transcription of ISG [83]. STAT1 from *Rousettus aegyptiacus* is highly conserved and, therefore, responsive to human IFNα [97]. Despite conservation of the signaling cascade, the IFN-I-dependent antiviral effector 2-5A-dependent endoribonuclease (RNASEL) is induced in *Pteropus alecto*, but not in humans [98]. In the same vein, multiple paralogs of the antiviral ISG BST2 and unique expression of STEAP4 (six-transmembrane epithelial antigen of prostate) upon IFN-I stimulation has been observed only in the microbat *Myotis daubentonii*, but not in humans or in the megabat *Pteropus vampyrus* [99]. Thus, bat-specific ISGs likely representing unique features of bat IFN systems have been uncovered. Taken together, genetic adaptations of IFN system and unique ISGs inductions in bats likely contribute to efficient control of viral infections and thus to disease resistance. Whether IFN-I abundance and kinetics hampers tissue repair, as shown in experimental mouse infection [100], potentially lowering disease tolerance, remains to be investigated.

#### 3.1.4. Type II interferons (IFN-II)

IFNγ is the only member of type II IFN family. It is produced by innate and adaptive lymphocytes and is crucial for host defense against intracellular pathogens [83]. *Pteropus alecto* IFNγ is closely related to other mammalian counterparts and is induced in splenocytes upon mitogenic stimulation. It has been shown to display antiviral activity against Semliki forest virus and HeV [101]. The antiviral activity has been also observed for *Rousettus aegyptiacus* IFNγ in the context of filoviruses [102]. *Pteropus alecto* IFNγ is under positive selection [62], and this finding has been extended to other bats by a comprehensive analysis, including 37 bat genomes [69]. However, whether the positive selection of IFNγ is linked to increased antiviral functions and/or decreased immune pathology in bats needs to be further investigated.

IFNγ-activating PYHIN members are lost in bats [62,69]. Similarly, the IFNγ-inducible related GTPase genes (IRGM1, IRGM2, IGTO, IIGP, and TGTP2) are lost in bat genomes [62,69]. Several reports have shown that IRGM negatively regulates cGAS and RIG-I induced IFN-I response and deficiency of IRGM leads to enhanced virus restriction, including SARS-CoV-2, Chikungunya virus (CHIKV), and Zika virus (ZIKV) [103,104]. Loss of IFNγ-inducible IRGM1/2 may result in elevated antiviral immunity in bats, but this requires verification. Absence of large families of IFNγ-inducible genes in bat genomes may represent a general mechanism to control the immunopathology driven by IFNγ in these mammals.

#### 3.1.5. Type III interferons (IFN-III)

IFN-III family comprises IFNλ1, IFNλ2, and IFNλ3 in humans, which are mainly expressed in hematopoietic and epithelial cells [84]. In *Pteropus alecto*, 2 IFNλ loci (IFNλ1 and IFNλ2) closely related to mammalian IFN-λ genes have been identified [105]. Their expression is induced by viruses or viral proxies, and both of them show antiviral functions [105]. IFNλ has been also detected in cells from *Rousettus aegyptiacus* upon infection with EBOV and MARV [102], and it inhibits EBOV replication [106]. Upon binding to the type III IFN receptor (IFNλR) consisting of IFNλR1 and IL10R2, IFN-III activates JAK-STAT signaling and induces the transcription of ISG. In contrast to the wide expression profile of IFN-I receptors IFNAR1/2, IFNλR1 in mice and humans is mainly expressed in epithelial cells [84]. IFNλR1 in *Pteropus alecto* is widely expressed in various tissues, and IFNλ2 treatment triggers ISG expression in epithelial and immune cells [107]. Whether such a broad expression pattern of IFNλR1 represents a universal antiviral mechanism in bats requires further investigations. The contribution of IFN-III signaling to disruption of epithelial barrier function and tissue repair in bats as demonstrated in virus-infected mice [100], and, thereby, a role in disease tolerance remains to be established.

### 3.2. Restrictive inflammasome activation and lack of short pentraxins

Inflammasomes are multiprotein oligomers that are initiated upon sensing danger-associated molecular patterns (DAMP) or pathogen-associated molecular patterns (PAMP) by cytosolic PRR [108]. Such PRR include nod-like receptors (NLR), Pyrin, AIM2, and IFI16. Upon activation, they assemble with the adaptor protein apoptosis-associated speck-like protein containing a caspase recruitment domain (CARD) (ASC) and caspase-1 to promote the activation of caspase-1. Active caspase-1 generates bioactive IL-1β and IL-18 [108], as well as N-terminal fragments of the Gasdermin D (GSDMD), which form membrane pores and mediate a lytic cell death termed “pyroptosis” [108].

**Fig 2:**
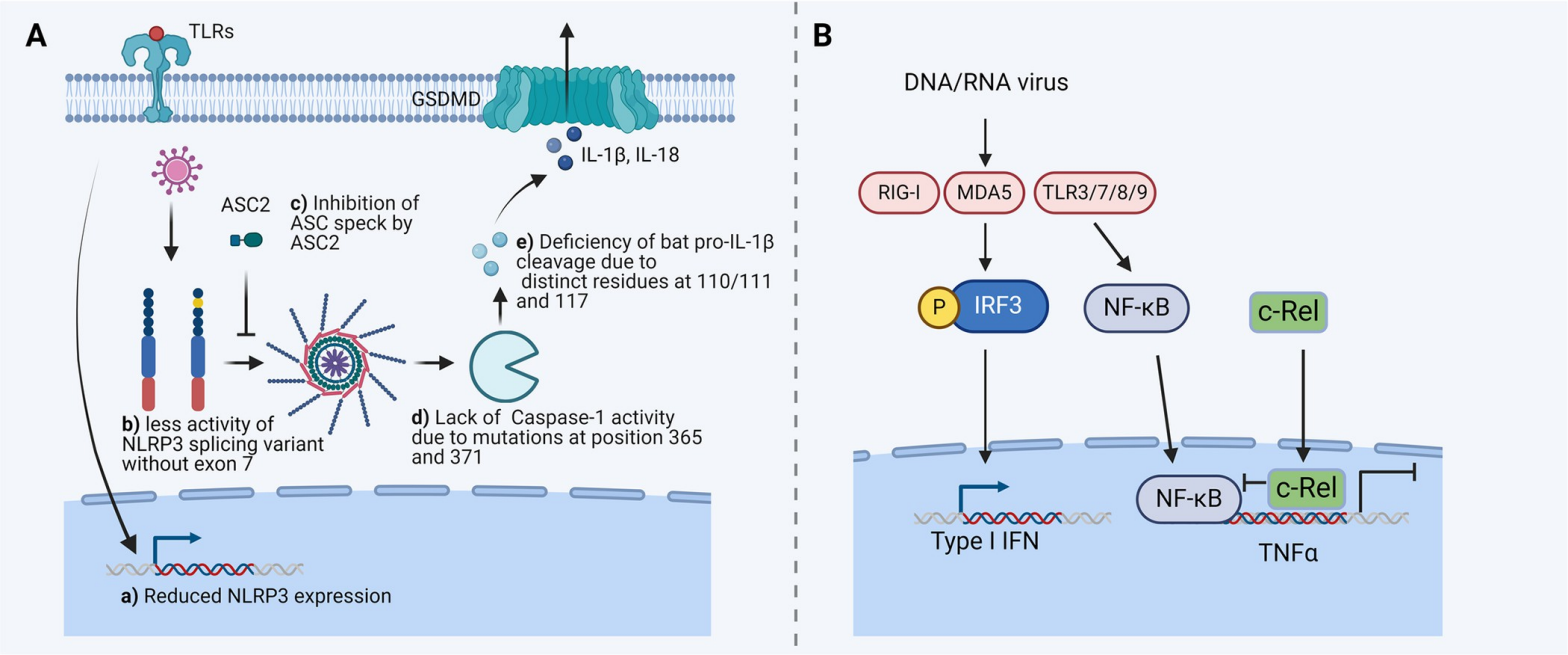
Dampened inflammasome activation and TNF-α production upon virus infection in bats. **(A)** Impaired inflammasome activation by various mechanisms in bats. Reduced NLRP3 expression upon priming, compromised NLRP3 activity due to splicing variants, lack of caspase-1 activity, and loss of pro-IL-1β cleavage have been described. ASC2 in bats is shown to inhibit the formation of ASC speck and inflammasome activation. (**B**) Limited TNF-α production in bats. TNFα expression is strictly regulated in cells from *Eptesicus fuscus* possibly due to unique c-Rel-binding motif in the promoter region of bat TNF-α. The figure was generated using the illustration software BioRender (BioRender.com). ASC, apoptosis-associated speck-like protein containing a CARD; cGAS, cyclic GMP-AMP synthase; GSDMD, Gasdermin D; IL, interleukin; IRF, interferon regulatory transcription factor; MDA5, melanoma differentiation-associated protein 5; NF-κB, nuclear factor kappa-light-chain-enhancer of activated B cells; NLRP3, NACHT, LRR, and PYD domains-containing protein 3; RIG-I, retinoic acid-inducible gene I; TLR, Toll-like receptor; TNF-α, tumor necrosis factor α.

Abundance of IL-1β and/or IL-18 correlates with severity of diseases caused by EBOV, SARS-CoV, and MERS in humans [11]. Besides loss of AIM2- and IFI16-mediated inflammasomes in bats (see section 3.1.2), activity of other types of inflammasome is diminished in bats. The NLRP3 inflammasome requires 2 steps encompassing priming and activation for generation of bioactive cytokines. In bats, NLR family pyrin domain containing 3 (NLRP3) is under positive selection [62], and its activation leads to reduced IL-1β and limited pyroptosis in *Pteropus alecto* in comparison with murine and human counterparts [20] (Fig 2A). Despite reduced inflammation the replication of IAV, Pteropine orthoreoviruses PRV3M and MERS-CoV in bat cells are comparable to murine and human cells, suggesting that impaired inflammasome activation is not due to defective virus replication and possibly uncoupled from antiviral immunity [20]. Reduced NLRP3 expression upon priming, NLRP3 exon 7-negative splicing variants, as described for *Pteropus alecto* and *Myotis davidii*, and reduced NLRP3 activity cumulatively contribute to dampened NLRP3 inflammasome activation in several bat species [20]. Validation in cells from other bats is required for extrapolating these observations to the entire Chiroptera order.

ASC contains an N-terminal PYRIN domain, which associates with inflammasome sensors to form oligomers, and a C-terminal CARD domain mediating caspase-1 activation [109]. Unlike ASC, ASC2 has only a PYRIN domain without a CARD domain. ASC2 was found exclusively in primates [110], but recently, it has been identified in several bat species from both Yinpterochiroptera and Yangochiroptera suborders [21]. Its expression is low in human tissues, but high in monocytes/macrophages and dendritic cells from *Pteropus alecto*. In response to inflammasome stimuli, bat ASC2 perturbs ASC speck formation and inhibits NLRP1, NLRP3, AIM2, or NLR family CARD domain containing 4 protein (NLRC4) mediated IL-1β secretion and pyroptotic cell death [21] (Fig 2A). Using ASC2-transgene mice, a benefit of this negative inflammasome regulator on survival upon IAV, ZIKV, or PRV3M infection has been demonstrated [21].

The levels of proinflammatory IL-1β in bats are also fine-tuned by the activity of the caspase-1 [111]. The enzyme has 2 substitutions at amino acid positions 365 and 371 (D365N and R371Q) in *Pteropus alecto* and *Pteropus vampyrus*, which diminish its cleavage capacity [111]. Intriguingly, caspase-1 from *Myotis davidii*, a bat from the Yangochiroptera suborder, cleaves *Pteropus alecto* pro-IL-1β, but not *Myotis davidii* pro-IL-1β. Point mutations in *Myotis davidii* pro-IL-1β confer its decreased cleavage potential [111]. Thus, diminished caspase-1 function and reduced potential of IL-1β maturation are complementary mechanisms also leading to dampened inflammasome activation in bats [111] (Fig 2A). However, whether mutations in caspases-1 and pro-IL-1β exist in other bat species remains ill-defined and comprehensive analysis covering more bat species are required. Furthermore, whether noncanonical inflammasome activation mediated by caspase-4/5/11 is altered in bats remains unknown.

Pentraxins belong to a superfamily of proteins characterized by a conserved pentraxin domain at the C-terminal and consist of 2 groups: short pentraxins and long pentraxins [112]. The short pentraxins are comprised of C-reactive protein (CRP) and serum amyloid P component (SAP) [112]. CRP is an acute-phase protein mainly produced by the liver in response to IL-6 and, to a lesser extent, IL-1β and tumor necrosis factor α (TNF-α). In infection, the serum concentration of CRP can increase up to 10,000-fold [112]; it contributes to opsonization of pathogens, but its binding to damaged or dead host cells may enhance tissue injury. In a feed-forward loop, CRP promotes the production of proinflammatory cytokines and chemokines [112]. Genome analysis of *Rousettus aegyptiacus* has confirmed the complete loss of functional CRP and SAP, indicative of a host strategy to restrict acute inflammation [113,114]. Whether other bat species lost as well short pentraxins is unknown. In sum, deficiency of short pentraxins may reduce inflammation in bats during viral infection. Reduced inflammasome activation and absence of pentraxins may cumulatively contribute to disease tolerance by restricting immunopathology during viral infections in bats.

### 3.3. Limited TNF-mediated inflammation

TNF-α induces inflammation by activating the expression of other proinflammatory factors and by triggering different forms of cell death, i.e., apoptosis or necroptosis [115]. For instance, abundant TNF-α in Ebola patients leads to endothelial cell dysfunction and vascular damage, as well as to lymphocyte apoptosis [116]. In bats, TNF-α expression seems to be strictly regulated. Cells from *Eptesicus fuscus* express levels of IFNβ comparable to human cells when stimulated with viral mimics, but lower TNF-α transcripts. Interestingly, a unique c-Rel-binding motif in the promoter region of TNF-α has been identified in several bats (*Myotis davidii* and *Miniopterus natalensis*), but not in humans. c-Rel suppresses transcription of TNF-α [54] and is a gene under positive selection in bats [62]. Whether bat-specific mutations of c-Rel contribute to its suppressor activity remains elusive. In contrast, cells from the mouse-eared bats (*Myotis myotis*) and *Pteropus alecto* generate more abundant TNF-α than murine counterparts upon PRR stimulation or HeV infection [117,118]. Whether these discrepancies stem from usage of different cell types, bat species or stimulations needs to be addressed (Fig 2B). Roles of TNF-α in disease tolerance via inflammation control are thus unclear.

### 3.4. Expansion of putative inhibitory NK cell receptors and their ligands in bats

Natural killer (NK) cells are innate lymphocytes essential for defense against tumors and virus-infected cells. NK cells express 2 structural families of NK cell receptors: killer cell immunoglobulin-like receptors (KIRs) and killer cell lectin-like receptors (KLRs), which initiate activating or inhibitory signals [119]. In *Pteropus alecto* and *Myotis davidii* genomes, functional KIRs and KLRs are lost [62]. A more comprehensive analysis has confirmed the loss of these receptors across the order Chiroptera [69]. Instead, *Pteropus alecto* and *Rousettus aegyptiacus* express an enlarged array of CD94 (cluster of differentiation 94)/NKG2 (natural killer group 2) (KLRD1/KLRC) lectin-like receptor heterodimers [86,120]. Of note, CD94/NKG2A heterodimeric receptor represents the major NK inhibitory receptor in humans and mice [121]. The majority of the putatively functional NKG2A/B-like genes in *Rousettus aegyptiacus* encode both immunoreceptor tyrosine-based inhibition motifs (ITIMs) at the cytosolic tail and positively charged residues in their transmembrane domains, indicating that they may function as inhibitory receptors. The MHC class I genes are also expanded, which could provide better self-recognition. Although none of them resemble HLA-E, the ligand of CD94/NKG2A, some of them are putative ligands conferring NK cells higher activation thresholds [86]. Unlike NKG2A/CD94, the activating receptor NKG2D forms homodimers and triggers downstream signaling via the adaptor molecule DNAX-activating protein of 10 kDa (DAP10) upon recognition of diverse ligands, such as MHC class-I-chain-related protein A (MICA) and MICB, retinoic acid early transcript 1 (RAET1), H60 and UL16-binding protein-like transcript 1 (Mult1) in mice, and the UL16-binding proteins (ULBPs) or RAET1 proteins in humans [122]. Interestingly, both RAET1 and H60 are absent in bats, possibly resulting in impaired activation of NK cells [69]. Single-cell RNA-seq of *Rousettus aegyptiacus* leukocytes and lung cells from the cave nectar bat *Eonycteris spelaea* has identified various clusters of NKT-like cells or NK/T cells, respectively [123,124]. Alterations of NK cell receptor repertoire and their ligands in bats may contribute to the impaired inflammation during viral infection and their role in disease tolerance awaits clarification.

### 3.5. Enhanced autophagy to reduce inflammation and/or restrict virus replication

Autophagy is an evolutionarily conserved process by which cytosolic components are targeted into double-membrane vesicles and subsequently degraded by fusing with lysosomes. Autophagy also functions as an innate defense mechanism against various intracellular bacteria and viruses [125]. Compared to human cells, *Pteropus alecto* cells display a higher level of basal autophagy and further heightened autophagy upon Australian bat lyssavirus (ABLV) infection [126]. The enhanced autophagy likely confers more efficient restriction of ABLV replication in bats [126]. Bats seem also to maintain an elevated autophagy especially upon serum starvation, which may support proteostasis and promote longevity [126]. Of note, 26 out of 70 autophagy-associated genes are specifically up-regulated in *Myotis myotis* bats, but down-regulated in humans and mice. Additionally, 10 of them are under positive selection in bat species, reflecting high evolutionary pressure on autophagy pathway [127]. The LC3II/I ratio, which is a marker of autophagy, significantly increases with age in *Pipistrellus kuhlii* and *Myotis myotis* bats, but not in mice, suggesting age-related up-regulation of autophagy in bats [127]. Considering that autophagy negatively regulates inflammation, especially inflammasome activation and IFN-I response [125], it is enticing to speculate that elevated autophagy in bats may lead to reduced inflammation and/or enhanced control of selective viruses.

## 4. Disease tolerance in bats

The limited development of clinical signs in bats infected with high-impact viruses (Table 1) strongly suggests that disease tolerance is operative in these reservoir hosts. Particularly the pattern of the inflammatory responses indicate that bats may minimize the impact of the infection on the host fitness. The mechanisms underlying disease tolerance have been recently summarized and networks of evolutionary conserved stress and damage responses regulators have been outlined [28]. We critically evaluate which mechanisms apply to bats and discuss evidence for disease tolerance in bats. We emphasize that bats hardly show disease symptoms upon infection with HeV [13], NiV [14], or MARV [22,114], although tissue lesions and virus replication have been detected in infected animals. Accordingly, they are symptom-free virus shedders. They resemble the infamous case of typhoid Mary, a healthy carrier of typhoid fever and an example of disease tolerance [25].

### 4.1. Mechanisms of disease tolerance

The mechanisms of disease tolerance that have been experimentally documented so far include the following: (i) neutralization of virulence factors and host detoxification; (ii) mitigation of cell stress; (iii) metabolic host adaptation and inflammation control; and (iv) tissue damage control and tissue repair [25,28]. Rapid neutralization of pathogen’s virulence factors, such as bacterial toxins, and efficient production and activity of detoxifying enzymes limit tissue injury. Bats primarily harbor viruses (see section 2), and these may employ, for instance, proteases to trigger infection and thus sustain the infectious process. For instance, 3C proteases or 3CL-like proteases from SARS-CoV-2 are required for viral replication and, in addition, cleave NLR members, such as NLRP1, to initiate inflammation [128]. Whether bat cells neutralize such viral factors more efficiently to limit tissue injury is unknown. From the host side, detoxifying enzymes alleviate accumulation of host deleterious antimicrobial byproducts. Heme oxygenase 1 (HO-1) [129] and xenobiotic receptors such as the aryl hydrocarbon receptor (AhR) [130] are examples of detoxifying factors that contribute to host fitness by conferring cytoprotection. MARV protein VP24 is shown to interact with human or bat Kelch-like ECH-associated protein 1 (KEAP1), leading to the activation of Nuclear factor erythroid 2-related factor 2 (NRF2)-mediated cytoprotective antioxidant response [131]. Whether these factors represent disease tolerance factors in bats remain to be elucidated.

Metabolic adaptations underlying disease tolerance encompass anorexia of infection, which directly affects the glucose tissue level, and the remodeling of the energy metabolism [132,133]. Experimental evidence from mouse models indicates that anorexia is protective in bacterial, but deleterious in viral infection [132,133]. There is only limited data on such behavior in bats, but recent experimental studies indicate that some bats do show anorexia upon experimental challenge [134,135]. *Rousettus aegyptiacus* develop anorexia, weight loss, social withdrawal, and lethargy upon endotoxin challenge [136]. On the contrary, the vampire bat *Desmodus rotundus*, despite decreasing overall activity levels in same conditions, do not show food intake changes and thus presumably no anorexia [137,138]. To what extent anorexia is present in distinct bat species during acute infection with zoonotic viruses is not well known; however, we did not observe this in *Rousettus aegyptiacus* upon experimental challenge with SARS-CoV-2 or influenza virus [41,139].

Resistance to infection requires extensive energetic costs due to immune activation and has been linked to catabolic metabolism. Disease tolerance has been connected to anabolism [140]. In conjunction with the capacity of bats for powered flight, which is unique among mammals, accelerated metabolism expenditure while flying has likely forced prioritization of energy for flight. Adaptation to flight has been associated with genetic imprinting of the bat immune system and trade-offs for high metabolic rates and longevity [141]. Accordingly, host defense necessitating less energy expenditures to maintain the physiological demands, notably disease tolerance over resistance, may be advantageous for bats. Such a metabolic-driven stratagem may enable bats to maintain viruses, yet further experimental proof for these associations awaits demonstration.

Mitigation of the cell stress is central to tissue damage control. A network of transcriptional factors and transcriptional regulators modulate activity of genes associated with stress responses and tissue remodeling [28]. Hierarchical structures have been estimated based on knowledge gained mostly from murine experimental models [28]. Master transcriptional regulators encompass molecules involved in cell proliferation (p53, CREB1 (CAMP responsive element binding protein 1)), hypoxia or oxidative responses (HIF-1α (Hypoxia-inducible factor 1-alpha), NRF2 (Nuclear factor erythroid 2-related factor 2)), enzymatic activity and metabolism (SIRT1 (Sirtuin 1), FOXO1 (Forkhead box protein O1), SREBF1 (Sterol regulatory element binding transcription factor 1)) or xenobiotic and proteotoxic stress (AhR, HSF1 (Heat shock factor 1), ATF4 (Activating transcription factor 4)) to name a few. Regulatory targets include HO-1, FAS (Fas cell surface death receptor), VEGFA (Vascular endothelial growth factor A), and PTGS2 (Prostaglandin-endoperoxide synthase 2), whereas CCDN1 (Cyclin D1), FOS (Fos proto-oncogene, AP-1 transcription factor subunit), or PDX1 (Pancreatic duodenal homeobox-1) act as repressors. A large proportion of genes in the DNA repair pathway, including Ataxia-telangiectasia mutated (ATM), DNA-PKC, RAD50, KU80, and MDM2 (Murine double minute 2), is under positive selection in bats, possibly minimizing the negative effects of the DNA damage [62]. More research on stress responses in bats and evaluation of expression and functionality of such networks postulated for other mammals should be a priority for understanding similarities between bats and other mammals with regard to disease tolerance molecular networks.

Capacity to limit inflammation has been demonstrated in certain bats, and this likely represents a major mechanism driving disease tolerance. Both unresponsiveness and skewed responses to pathogens can restrain inflammation. Immunological ignorance occurs when PRR sensing of pathogens are absent or dysfunctional, or when particular signaling pathway are blunted or constitutively switched off. Bats do lack PYHIN members, including AIM2 [68], which is a critical inflammasome sensor for cytosolic DNA. Due to the highly conserved serine residue (S358) in bat STING, cytosolic DNA-induced cGAS/STING signaling is also probably dampened in bats [75].

Inflammation can also be controlled by regulating abundance of inflammatory mediators. Levels of proinflammatory IL-1β released in response to canonical inflammasome activators in bat cells are reduced compared to those detected in human or murine systems [20,111], and bat ASC2 negatively regulates various inflammasomes by perturbating ASC complex formation [21]. This may explain scant inflammation in *Rousettus aegyptiacus* and *Rousettus leschenaulti* upon experimental challenge with coronaviruses or MARV [139,142]. As discussed already, limited inflammation has been also connected to the absence of short pentraxin, possibly CRP, at least in *Rousettus aegyptiacus* challenged with MARV or EBOV [113,114]. These immune response patterns largely differ from their human counterparts. Absent induction of inflammatory mediators, such as CCL-8 or IL-6, has been unveiled in monocytes from MARV-infected *Rousettus aegyptiacus* [22]. However, diminishment of proinflammatory responses in *Rousettus aegyptiacus* has led to increased MARV replication, shedding, and pathology, suggesting that proinflammatory responses also contribute to the controlling of MARV [143].

Limitation of the tissue damage is intertwined with inflammation and central to disease tolerance. Immune cells with immune regulatory features are critical for repair or maintenance of homeostatic tissue function. Regulatory T cells (Treg) and M2-like macrophages have been assigned such roles [28]. Recent studies in SARS-CoV2-challenged *Artibeus jamaicensis* have identified Treg-like cells in hACE2-transduced bats showing pathology, but no symptomatology [144]. A strong bias toward the Th17 and regulatory T cell subsets within the splenic CD4^+^ T cells has been noted in *Pteropus alecto* at steady state [145]. While a bias toward M2-like CD206-expressing myeloid cells has been detected in *Rousettus aegyptiacus* upon age progression [124], this bat species also shows an M1- to M2-like early transition of macrophages upon infection with filoviruses [114]. Switching on regulatory cells of lymphoid and myeloid origin may dampen inflammation and also drive tissue repair. Cytokines involved in tissue regeneration, notably IL-6, IL-22, and TNF-α, have been measured in few bats species, and results do not indicate abundance upon infection. Since these are also inflammatory factors, specific detection in cell subsets would be informative. TGF-ß transcripts in putative Treg cells in SARS-CoV-2-infected *Artibeus jamaicensis* and IL-22 transcripts in healthy *Pteropus alecto* have been detected [145,146]. Regeneration of barrier tissue is modulated by IFN, and recent reports indicate that particularly IFN-λ reduces epithelial proliferation and differentiation [147]. Since certain IFN-I are constitutively expressed in some bats (see sections 3.1 and 3.3), it will be critical to evaluate effects of IFN-I and IFN-III on tissue repair in context on the disease tolerance in bats.

Besides established molecules and pathways, newly reported determinants of disease tolerance should be included in investigations. For instance, epigenetically driven mechanisms [148] should be investigated in bats given the universality of the epigenetic marks across mammals. The microbiome has been reported to shape host response toward disease tolerance, and it is driven by either bacterial metabolic products such as short chain fatty acids or D-lactate, enzymes such as bacterial fucosidase, or selective activation of PRR or oxidative stress responses, for instance, NRF2 [149]. Whether the microbiome also impacts on disease tolerance in bats requires to be elucidated.

**Fig 3:**
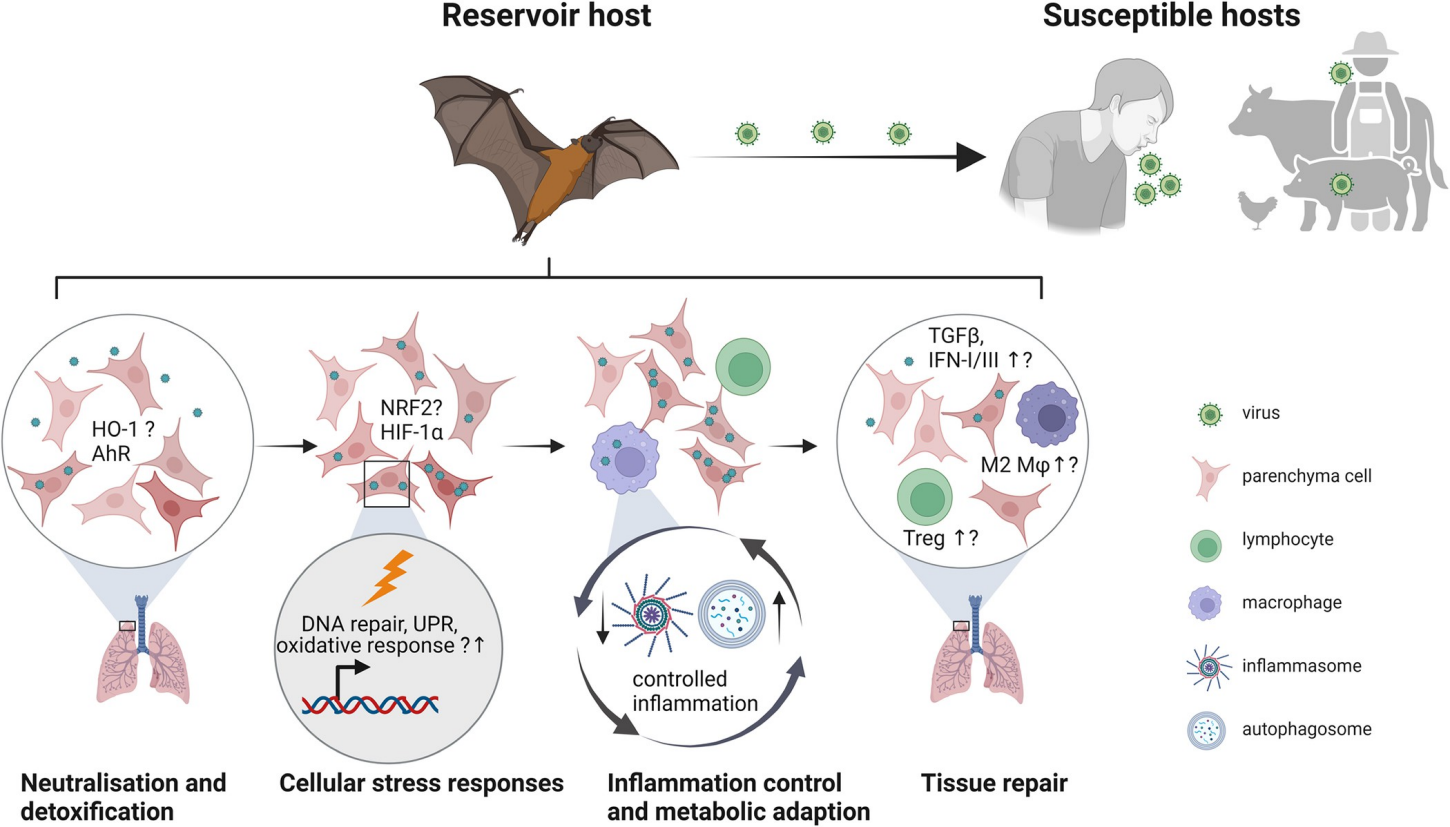
Disease tolerance mechanisms operating in bats infected with high-impact, zoonotic viruses. Bats infected with viruses lethal to other mammals remain usually asymptomatic. Their disease tolerance may stem from effective host detoxification and neutralization of viral effectors, mitigation of the cell stress, development of a blunted inflammatory response, and an efficacious tissue repair. While most evidence support restriction of inflammation in bats, involvement of master regulators for cell stress ad tissue damage remain elusive. The figure was generated using the illustration software BioRender (BioRender.com). AhR, Aryl hydrocarbon receptor; DC, dendritic cell; HIF-1α, hypoxia-inducible factor 1-alpha; HO-1, Heme oxygenase 1; IFN, interferon; Mφ, macrophage; NRF2, nuclear factor erythroid 2-related factor 2; TGF-β, transforming growth factor beta; Treg, regulatory T cells; UPR, unfolded protein response.

In summary, disentangled viral replication from inflammation [20], genomic traits including deletion of gene family with proinflammatory roles [86,113], and a bias toward regulatory cells [124,146] substantiate reduced inflammation in bats. These observations strengthen the evidence that inflammation control rather than immune activation characterizes responses to viruses in bats. However, sparse evidence substantiates adoption of other disease tolerance mechanisms in bats (Fig 3).

### 4.2. Disease tolerance in defense against and persistence of viruses

To evaluate universality of disease tolerance in bats, it must be established whether various virus families, bacteria, and fungi can establish infection while maintaining the host fitness. Most bat-borne viruses seem to have this capacity. Although ACE2 from both *Tadarida brasiliensis* and *Rousettus aegyptiacus* are able to mediate the entry of SARS-CoV2 [150,151], SARS-CoV2 experimental infections in these bats have led to lack of disease signs and inconsistency regarding replication and shedding of the virus [139,152,153]. Similarly, bats seem to tolerate MARV, HeV, and NiV [13,14,22,114]. In all these examples, bats do survive infection with limited or no sickness (Table 1). In contrast, experimental infections with different lyssaviruses cause severe or even fatal diseases in bats (Table 1), although serological field studies have demonstrated the presence of lyssavirus neutralizing antibodies in healthy bats, indicating that bats may overcome a lyssavirus infection. The underlying mechanism regulating the pathogenesis and outcome of lyssavirus infection in bats is still poorly understood [154]. What makes the bat host reacting differently? One potential explanation could be the intrinsic damage susceptibility or variable repair capacity of affected host tissues. Brain tissue and neurons have limited regenerative capacity and are very sensitive to damage [25]. Thus, the tropism of the lyssaviruses and the low disease tolerance of the brain tissue support discrepant disease features of the mentioned zoonotic viruses in bats. Zoonotic viruses belonging to Filoviridae, Paramyxoviridae, and Coronaviridae often infect the respiratory and digestive tract. The functional anatomy of cells within such tissues may differ, and various respiratory and digestive organs may variably respond to tissue damage with regard to tissue sequelae. Endothelial damage has severe consequences in humans infected with EBOV. Whether and how bat endothelia turn on reparatory pathways promptly after EBOV infection is unknown. Vasodilatation and antithrombotic events may limit severe damage in EBOV-infected *Rousettus aegyptiacus* [114]. Whether pathogens that kill certain bat species, such as fungi, circumvent resistance mechanism and, at the same time, hinder disease tolerance has not been elucidated. Selectivity of disease tolerance for particular bat species and a given pathogen class cannot be excluded. Further, whether viruses themselves benefit from disease tolerance in bats and whether viral pathogens actively drive disease tolerance for persistence in bats has not been established so far.

Disease tolerance is effective at maintaining the host fitness in spite of high pathogen burdens. This comes at a cost: It confers the host with reservoir abilities and favors spread of the infection at the population level [28]. As such, the individual fitness may impact on the epidemiological status of the entire population. At the population scale, disease tolerance may be beneficial, but it does contribute to microbial persistence. Bats are natural reservoirs for zoonotic viruses; thus, the question of how disease tolerance in bats enable viral persistence must be addressed. Persistence in a given individuum has been repeatedly documented for MARV [22,155], but causal links to specific tolerance mechanisms await demonstration. Persistence in a population may be due to ecological as well as immunological factors. Serology studies in wild-caught *Eidolon helvum* have suggested that some bats experience prolonged infectious periods or within-host latency [156]. Age-dependent variability in immune composition, including enrichment for putative regulatory components [124], may condition higher frequencies of coronaviruses in juvenile *Pteropus lylei* [157] and birth-related pulses of MARV spillovers from *Rousettus aegyptiacus*, and risks for human infection [158]. Large-scale field observations and challenge infections in diverse populations with respect to age, sex, nutritional and physiological status should be pursued to clarify the impact of disease tolerance or the carrier status of bats.

## 5. Perspectives

Bats are unique among mammals in many ways, including their immune response, as highlighted already. Despite the abundant and diverse bat virome, the majority of bat viruses have not emerged to cause disease in other mammals, humans or livestock, which calls for a balanced view. While considering their reservoir role, many bat species are considered threatened by the International Union for the Conservation of Nature (IUCN). In multiple ecosystems, bats are required for pollination, seed dispersal, and plant pest control [159]. Their roles in ecosystem maintenance must be thus prioritized, and universal associations with dangerous viruses should be cautioned.

While discussing bat-borne viral pathogens, bat immune responses, and disease tolerance, it has become obvious that we are left with more questions than answers. We provide a catalogue of outstanding questions specific for disease tolerance and suggest experimental approaches to address them. For infection biologists, probably the most burning question remains how bats allow asymptomatic viral replication? Is disease tolerance (equally) operating in bats considering their diversity in ecology, physiology, and social behavior? Most of the immunological observations come from few bat species, namely, black flying foxes (*Pteropus alecto*), Egyptian fruit bats (*Rousettus aegyptiacus*), cave nectar bats (*Eonycteris spelae*), mouse-eared bats (*Myotis* spp.), Jamaican fruit bats (*Artibeus jamaicensis*), and horseshoe bats (*Rhinolophus* spp.), and it remains a challenge to extrapolate findings to all bat species. Various bats have different dietary habits and likely diverse microbiomes. Do such differences have an impact on disease status in different bats species? Disease tolerance should be evaluated across various bat species using the same fitness. Do life-history traits train bat’s immune system to deploy more effective antimicrobial responses and resist or tolerate specific infections? Is there any evidence for selective fixation of fitness traits? How do bat immune features related to dampened inflammation arise? Long-term field studies are necessary to answer these questions. Moreover, an inventory of potential tolerance mechanisms should be compiled across various bats as novel determinants of disease tolerance could be unique to certain bat species. Is the disease tolerance in bats universal, i.e., irrespective of virus and bat species? Certainly not, as differences in outcome of disease and species susceptibility have been documented. Are bats adapted to tolerate all viral infections or only those they have coevolved with? Here, epidemiology and ecoimmunology, as well as targeted laboratory studies, would be informative. Field studies extending over decades have recently provided evidence that bat ecology drives spillovers of HeV from Pteropodid bats [160]. Nutritional and energetic stress has led to distinct land usage, and such stressors may impact also on patterns of immune responses. Resources trade-offs may tip the balance toward disease tolerance over resistance as this mode of defense is less energy demanding.

Host immune responses impose pressure on pathogens and drive microbial evolution. Does disease tolerance reduce pathogen pressure and does it select stable microbial phenotypes? It is postulated that reservoir hosts have higher pathogen circulation and infection periods than nontolerant hosts, which results in a genetically diverse pool of pathogens. If this is the case, is a spill over event serendipity-driven or conditioned by the host, for instance, an immune status allowing heightened microbial growth and further evolution in an intermediate host? We have discussed existing lines of evidence for disease tolerance and propose that substantial experimental proof is awaiting. We envisage that there is no “one size fit all” scenario for all bats and all pathogens they harbor. The catalogue of questions we have presented is not exhaustive and as disease tolerance is unfolding more questions will arise for bats, too.

The bat immune system is poorly characterized relative to potential medical gains from these animals. We may learn from bats how to tolerate rather than resist disease and thus to reduce the virus-triggered damage rather than aiming to eliminate the pathogen at high costs for the host. The study of bats as nonmodel organisms should be supported by new technologies that overcome, to some extent, paucity of immunological exploratory tools such as monoclonal antibodies for cell phenotyping. In a further step, integrative ecoimmunology can be implemented, as well as usage of computer-based tools and artificial intelligence. Computational models for human and bat immune responses to viruses were recently developed [161]. Knowledge of disease tolerance from murine models along with experimental challenge-derived multiomics data have been trained in models to generate “disease space” maps [162]. Such topological network maps would first help define trajectories of the infection and second define contribution of resistance and tolerance to infection outcome. Iterative bioinformatic and experimental approaches may support identification of bat unique mechanisms of disease tolerance, if any, and also evaluate their potential therapeutic use. Computational and wet lab investigations in *Xenopus larvae* have been applied to discover host-directed therapies strategies for antibiotic-resistant bacteria [163], and similar approaches can be adapted to discover disease tolerance-driven antiviral pathways for emerging viral infections using bat-specific novel mechanisms. Disease tolerance is no longer at a nascent stage in immunology research. It should thus be incrementally considered and experimentally explored in bats. It may not be the “one size fit all” host defense strategy for bats, but one relevant to address in order to illuminate bat biology and devise innovative therapies.
